# In Situ Monitoring of the Curing of Highly Filled Epoxy Molding Compounds: The Influence of Reaction Type and Silica Content on Cure Kinetic Models

**DOI:** 10.3390/polym16081056

**Published:** 2024-04-11

**Authors:** Julian Vogelwaid, Felix Hampel, Martin Bayer, Michael Walz, Larysa Kutuzova, Günter Lorenz, Andreas Kandelbauer, Timo Jacob

**Affiliations:** 1 Mobility Electronics, Engineering Technology Polymer & Packaging, Robert Bosch GmbH, 72770 Reutlingen, Germany; fixed-term.felix.hampel@de.bosch.com (F.H.); martin.bayer2@de.bosch.com (M.B.); michaeldavid.walz@de.bosch.com (M.W.); 2 Fakultät für Naturwissenschaften, Institut für Elektrochemie, Universität Ulm, 89081 Ulm, Germany; timo.jacob@uni-ulm.de; 3 Fakultät für Life Sciences, Reutlingen University, 72762 Reutlingen, Germany; larysa.kutuzova@reutlingen-university.de (L.K.); guenter.lorenz@reutlingen-university.de (G.L.); andreas.kandelbauer@reutlingen-university.de (A.K.); 4 Institute of Wood Technology and Renewable Materials, University of Natural Resources and Life Sciences, 1180 Vienna, Austria

**Keywords:** dielectric analysis (DEA), epoxy molding compound (EMC), kinetic modeling, time–temperature–transformation (TTT) diagram, glass transition temperature (*T*_g_)

## Abstract

Monitoring of molding processes is one of the most challenging future tasks in polymer processing. In this work, the in situ monitoring of the curing behavior of highly filled EMCs (silica filler content ranging from 73 to 83 wt%) and the effect of filler load on curing kinetics are investigated. Kinetic modelling using the Friedman approach was applied using real-time process data obtained from in situ DEA measurements, and these online kinetic models were compared with curing analysis data obtained from offline DSC measurements. For an autocatalytic fast-reacting material to be processed above the glass transition temperature *T*_g_ and for an autocatalytic slow-reacting material to be processed below *T*_g_, time–temperature–transformation (TTT) diagrams were generated to investigate the reaction behavior regarding *T*_g_ progression. Incorporating a material containing a lower silica filler content of 10 wt% enabled analysis of the effects of filler content on sensor sensitivity and curing kinetics. Lower silica particle content (and a larger fraction of organic resin, respectively) favored reaction kinetics, resulting in a faster reaction towards *T*_g1_. Kinetic analysis using DEA and DSC facilitated the development of highly accurate prediction models using the Friedman model-free approach. Lower silica particle content resulted in enhanced sensitivity of the analytical method, leading, in turn, to more precise prediction models for the degree of cure.

## 1. Introduction

Epoxy resin molding compounds are thermosets with excellent chemical and mechanical properties, as well as good adhesion to printed circuit board (PCB) material and copper. Electrical insulation plays a key role in electronic packaging using thermosets. Therefore, thermoset materials are highly filled with up to 90 wt% silica particles [1,2]. In the electronics and microelectronics industry for semiconductor devices and microchips, these materials are ideal as packaging materials for integrated circuit boards, hybrid circuit boards, and transistors. The main epoxy molding compound (EMC) processing methods are injection molding, compression molding, and transfer molding [1,2,3,4].

EMCs often contain a substantial number of micron-sized silica particles to enhance the stiffness and strength of epoxy molding products. Silica particles improve thermal and electrical isolation [5,6,7] and increase the Young’s modulus, resulting in reduced elongation of the composite material [6]. Careful selection, optimal quantity, and proper integration improve their durability and mechanical properties [8]. On the other hand, the cross-linking reaction can be enhanced or reduced by incorporating fillers into the thermoset formulation, depending on the interaction between the polymer and filler. To improve the bonding between fillers and matrix, the surface of the fillers can be coated with functional silane as a coupling agent. Consequently, an increased reaction rate with increasing filler content is achieved by catalyzing the epoxide amine reaction and through interfacial effects [9]. However, the reduction in activation energy observed in the reaction shows no significant effect on autocatalyzed reactions in highly filled epoxy compounds [10]. On the other hand, the particle size of the filler influences the reaction kinetics. A reduction in filler particle size leads to a reduction in activation energy even at a filler content of 0.1 wt% [11]. The majority of studies on the influence of fillers on reaction kinetics, however, are based on relatively low filler contents, and the influence of filler size is also not systematically investigated [5,7,12].

In recent years, thermoset processing has evolved in the area of process control. An important part of molding is therefore the placement of various sensors in the cavities [3]. The data generated by sensors are utilized for process monitoring and can be used to detect the smallest deviations, and thus defective parts, at an early stage [13,14].

Monitoring temperature and pressure enables process stability to be controlled in terms of cavity filling and reaction temperature. Understanding the curing behavior of thermoset molding compounds in the area of the changing molecular weight throughout the entire process is crucial for achieving favorable molding results. During molding, the glass transition temperature (*T*_g_) and molecular weight increase. The progression of *T*_g_ depends on the kinetics of the materials and is significantly controlled by the temperature. If the reaction is carried out below the estimated ideal backbone glass transition temperature of the fully reacted system (*T*_g∞_), the *T*_g_ at a certain degree of cure at a specific temperature (*T*_g1_) of the polymer will reach the reaction temperature (*T*_cure_) *T*_g1_ = *T*_cure_ [15]. If the reaction takes place at temperatures above *T*_g∞_, *T*_g1_ is progressing according to the kinetic behavior. By combining kinetic data and *T*_g_ values, to a certain degree, cure time–temperature–transformation (TTT) diagrams can be derived.

TTT diagrams enable the observation of physical states throughout the curing process, serving as a tool for comprehending the curing mechanism of thermosetting resins [16,17,18]. To generate TTT diagrams for the resin system, information about the glass transition temperature (*T*_g_) and the degree of cure are required. The properties are usually determined by methods such as dynamic mechanical analysis (DMA), thermal mechanical analysis (TMA), differential scanning calorimetry (DSC), or rheological methods [19,20]. Based on such data, the vitrification behavior of the epoxy resin can be represented in a diagram in terms of temperature and time [17,18]. The TTT diagram provides information on possible applications for materials, cured to a certain degree [21].

Generating TTT diagrams for the respective EMC system involves the determination of *T*_g_ values for various degrees of cure. Using the DiBenedetto equation, the *T*_g_ values can be represented graphically over temperature and curing state [17,22,23]:

(1) $$\frac{{(T}_{g}-T_{g0})}{{(T}_{g1}-T_{g0})}=\frac{\lambda \alpha }{\left(1-\left(1-\lambda \right)\alpha \right)}$$

Here, *T*_g0_ is the glass transition temperature of the raw material in an uncured state, whereas *T*_g1_ is the glass transition temperature of the fully cured material. *λ* is a material fitting constant and influences the shape of the DiBenedetto curve. Correlating *T*_g_ and the degree of cure, the reaction kinetics of the respective materials can be used to determine and visualize the course of *T*_g_ as a function of temperature and degree of cure.

Temperature is one of the most important influencing factors during the entire molding process, as it substantially affects the reaction kinetics and *T*_g_ of thermosets [3]. The temperature influence begins with the preheating of the material before injection and continues via the preliminary viscosity state of the molding compound, which is generated by different injection speeds, until the isothermally tempered cavity is reached. Important parameters for this process are the thermal conductivity and the viscosity change during injection, including melting phase and the already initiated reaction [3,4,13,14,24,25].

In daily practice, it is a huge challenge to optimize the industrial process and ensure consistently high product quality. Direct process control during the encapsulation process is only possible using a large number of sensors. Real-time information on the cross-linking state of the material during curing cannot be derived from simple temperature and pressure sensors [26]. Thus, offline methods such as DSC, DMA, or TMA are widely used for determining the curing kinetics. The cross-linking conditions simulated with such instruments are, however, not directly comparable to those present in the cavities of direct packaging processes under real-life conditions [26,27,28,29,30]. Alternatively, online monitoring sensors such as Raman spectroscopy, infrared spectroscopy, ultrasonic monitoring, or dielectric analysis can, in principle, be used to determine the kinetics of the cross-linking process in real time, provided that suitable sampling strategies are available [31,32,33].

DEA offers huge potential for industrial use as a process control instrument, as it is robust compared to other sensor technologies, easy to incorporate into tools, and, most importantly, is associated with significantly lower costs [34]. In addition, DEA provides an advantage over optical methods because it is suitable for measuring opaque materials as well [31,32,35]. However, compared to the cure monitoring methods of spectroscopy and ultrasonic monitoring, DEA is dependent on temperature effects, including those caused by shearing [36,37]. Furthermore, spectroscopic methods have higher accuracy with respect to the beginning of the reaction, during melting, and at the end of the reaction, when ionic motion is already frozen by the existing polymer network [35].

A temperature dependence of the DEA signal, resulting from the thermodynamics of the ion movement of the induced temperature, decreases with rising temperature, since the ion mobility is restricted at a maximum comparable to a limited exponential behavior [19,20,38]. However, it was shown that the temperature effect can be eliminated by applying an empirical compensation factor [36]. This method derives from combining the equations describing the dependence of ion viscosity on temperature, expressed in terms of polymeric network resistance, the Einstein ratio, and the Arrhenius equation. The variables in these equations are combined to form empirical factors, and the measured DEA data are corrected to a certain temperature level. Using this correction, all calculated effects of temperature can be related to the target variables and are not dependent on the measurement technique by itself [36].

In this work, the effect of the silica particle content on the in-line calculated kinetics and its effect on the glass transition temperature of an epoxy material highly filled with silica particles is shown. The different calculated DEA kinetics of the epoxy compounds are compared with DSC kinetics at different heating rates and isothermal conditions.

## 2. Materials and Methods

### 2.1. Materials

**Figure 1:**
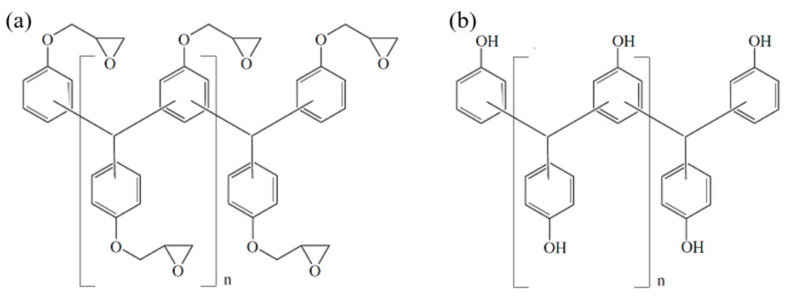
Multifunctional epoxy resin (**a**) and multifunctional phenolic hardener (**b**).

Two commercially available pre-mixed, latent-curing epoxy molding compounds (EMC) with a high filler content of about 83 wt% (EMC 1) and 73 wt% (EMC 2) spherical silica particles with a nucleophilic curing agent were used in this study. A third material (EMC 3) with a silica particle content of 81 wt%, classified as a fast-curing substance, was additionally examined. The basic chemical structure of a multifunctional epoxy resin is presented in Figure 1a and for a multifunctional phenol hardener is shown in Figure 1b. EMC 1 and EMC 2 exhibit a *T*_g_ of 220 °C in the fully cross-linked state, while EMC 3 has a *T*_g_ of 162 °C. At a processing temperature in the range of 165 to 185 °C, EMC 1 and EMC 2 are therefore processed at a temperature below their final *T*_g_, whereas EMC 3 is molded above its final *T*_g_. Each material was stored at 2 °C and warmed up to room temperature for >8 h prior to use.

### 2.2. Dielectric Analysis (DEA) in Transfer Mold

**Figure 2:**
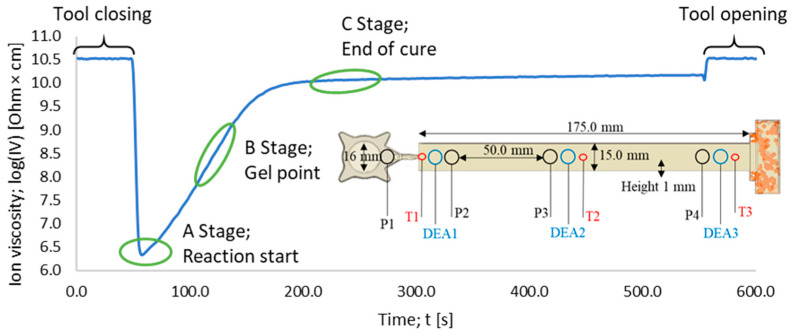
The DEA curve of a measurement at 175 °C is shown. The three reaction stages—the reaction start A, the gel point B, and the end of cure C—are labeled. The design and location of the inline sensors in the processing equipment are depicted. Thermocouple type K near the gate: T1; monotrode for dielectric analysis: DEA1.

Dielectric measurements were performed with a 4/3RC monotrode (NETZSCH-Gerätebau GmbH, Selb, Germany) and a temperature sensor thermocouple type K (Kistler Instrumente AG, Winterthur, Switzerland), which were connected to a DEA analyzer (DEA 288 Epsilon, NETZSCH-Gerätebau GmbH, Selb, Germany). The sensors were integrated into a slit mold cavity (175.0 × 15.0 × 1.0 mm), which was mounted on a transfer mold press. The location of the DEA and temperature sensors inside the mold’s slit die cavity are shown in Figure 2.

For further understanding, the DEA signal shown in Figure 2 needs to be interpreted on a molecular level. Usually, for thermosetting materials, different stages can be distinguished. Stage A describes the reaction’s start, where the degree of cure is zero. Stage B represents the maximum reaction rate, which can be determined as the peak of the derivation. Within stage B, ion movement is more inhibited due to the formation of polymer networks. Afterwards, in stage C, the material starts to build highly dense polymer networks. For stage C, the degree of cure is considered as one. This leads to maximum inhibition of ions during polymerization, causing the measured resistivity to reach a plateau and its peak value during the curing process [34].

**Figure 3:**
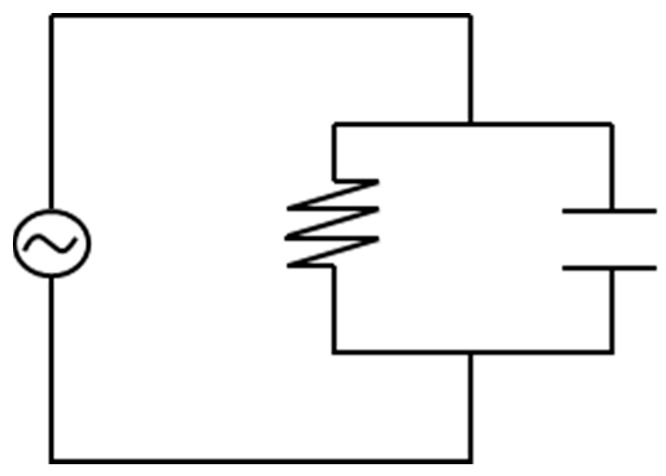
A regular circuit is shown with both an alternating current (AC) and a direct current (DC).

A dielectric measurement has, as a principle of measurement, a conductivity applied to a material, resulting in a measured resistivity. For polymers, the measured conductivity can be described by the contributors for alternating current (AC) and direct current in a regular circuit as shown in Figure 3 (DC) [34].

Both contributors can be expressed individually by the following correlations:

(2) $$\rho_{DC}-ion\;movement$$

(3) $$\rho_{AC}-rotating\;dipoles$$

For cross-linked polymers, it was found that the ion movement, with respect to ion viscosity (*IV*), is more reliable for gaining insights on the degree of cure [34] compared to rotating dipoles. Therefore, the resistivity is then expressed as follows:

(4) $$\rho_{DC}=IV=\frac{1}{\sigma_{DC}}=\frac{1}{q\mu n}$$

where${\;\sigma }_{DC}$ is the time-alternating conductivity (ohm^−1^·cm^−1^), *q* is the magnitude of electronic charge (coulombs), *μ*(*t*) is the free ion mobility (cm^2^/(V·s)), and *n* is the free ion concentration (cm^−3^) [34]. The value for the free ion movement is connected to the Stokes–Einstein equation, which expresses the resistivity as follows:

(5) $$\rho_{DC}=IV=\frac{k_{B}}{q^{2}nD_{0}}\cdot Te^{\frac{Q}{k_{B}T}}$$

where $D_{0}$ is the diffusion coefficient (cm^2^/s), *k*_B_ the Boltzmann’s constant (eV/K), *T* is the absolute temperature (K), and Q the heat quantity. By applying the natural logarithm, the DEA signal can be expressed as follows:

(6) $${log}_{10}\left(\rho_{DC}\right)=\log_{10}\left(IV\right)\;=\log_{10}\left(\frac{k_{B}}{q^{2}nD_{0}}\right)+\log_{10}T+\frac{E_{a}}{k_{B}Tln\left(10\right)}$$

**Figure A16:**
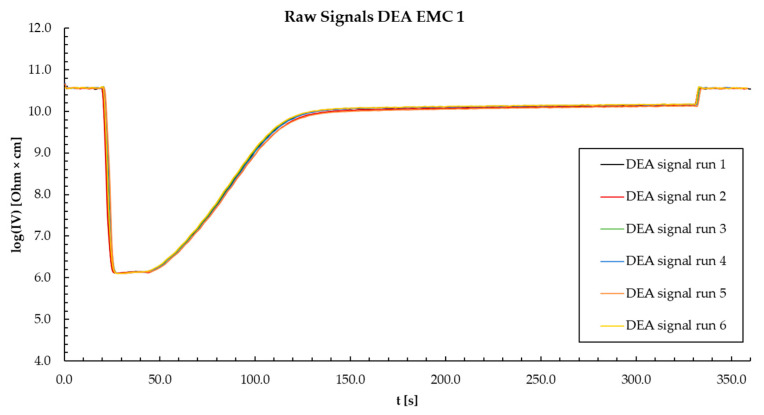
An example of raw data of the DEA measurements of material EMC 1 at a temperature of 175 °C and an injection speed of 2.5 mm/s. The raw signals of the 6 repeated measurements of sensor 1 are shown.

**Figure A17:**
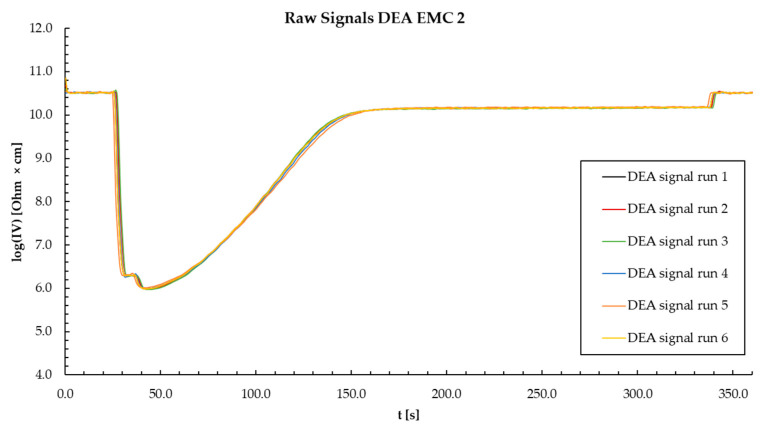
An example of raw data of the DEA measurements of material EMC 2 at a temperature of 165 °C and an injection speed of 2.5 mm/s. The raw signals of the 6 repeated measurements of sensor 1 are shown.

**Figure A18:**
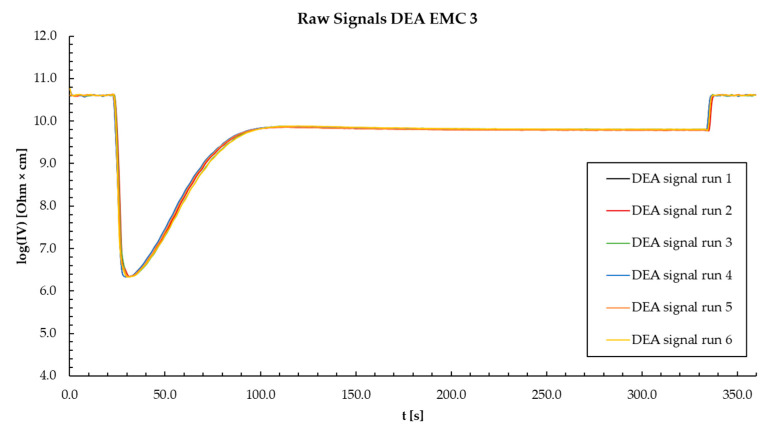
An example of raw data of the DEA measurements of material EMC 3 at a temperature of 185 °C and an injection speed of 2.5 mm/s. The raw signals of the 6 repeated measurements of sensor 1 are shown.

The experiments were performed with 10 and 100 Hz as measurement frequencies. First, the reproducibility of the DEA signal was checked with kinetic analysis trials, which were evaluated by performing 6 isothermal inline measurements at different temperatures (165, 175, and 185 °C) and at different injection speeds (1.0, 2.5, and 4.0 mm/s) with a recording time of 5 min. Figure A16, Figure A17 and Figure A18 in Appendix P, Appendix Q and Appendix R give examples of the reproducibility of DEA measurements for the materials analyzed.

### 2.3. Kinetic Analysis via Dielectric Analysis (DEA)

The model-free kinetic and the resulting kinetic model by Friedman has its origin in the Arrhenius equation:

(7) $$k=A\cdot e^{\frac{-E_{a}}{RT}}$$

To be able to use the Arrhenius equation to describe the dielectric measurement, two assumptions need to be considered [39]: First, the reaction can be described by a correlation between the degree of cure and the reaction constant [39]. Second, the reaction constant is a set value for conversion as a function of time [28,39]. The degree of cure can be defined as follows:

(8) $$\alpha =1-\frac{∆H_{t}}{{∆H}_{total}}$$

By considering the aforementioned assumptions, the relationship between the degree of cure α and the reaction constant k is expressed as follows:

(9) $$\frac{d\alpha }{dt}=k\cdot f\left(\alpha \right)$$

where f(α) is a function of the degree of cure versus time. This results in the following calculation:

(10) $$\frac{d\alpha }{dt}=A\cdot e^{\frac{-E_{a}}{RT}}f\left(\alpha \right)$$

By applying the natural logarithm afterwards, the general model-free kinetic equation is gained:

(11) $$\ln\left(\frac{d\alpha }{dt}\right)=\ln\left[f\left(\alpha \right)A_{\alpha }\right]-\frac{E_{\alpha }}{RT_{\alpha ,j}}$$

*T_α_*_,*j*_ is the temperature for the correlated degree of cure. For the kinetic investigations and for all ion viscosity values, data preparation is needed. In previous works, it could already be shown that a temperature adjustment is needed, due to the influence of the temperature on the ion viscosity itself. Therefore, two temperature coefficients were implemented [36].

(12) $${log}_{10}{\left(\rho_{10}\right)}_{norm}=A+\log_{10}\left(T_{norm}\right)+T_{measured}\log_{10}(\rho_{DC})\;+\left(c_{1}T_{measured}+c_{2}\right)\frac{1}{T_{norm}}$$

Applying the temperature coefficient, the values for the ion viscosity can be compared between measurements at different temperatures. The temperature dependence of the ion movement is therefore eliminated [36].

The deviation of the general kinetic equation based on the model-free kinetic model by Friedman is shown in Equation (11). This equation can be used for modelling; therefore, the values for alpha, time, activation energy, and temperature need to be determined [39].

Alpha can be gained by taking the maximum and minimum values for the ion viscosity and determining them as the values for the minimum and maximum degree of cure [39,40].

(13) $$IV_{max}=\alpha_{max}=1;IV_{min}=\alpha_{min}=0$$

An integral form of the kinetic expression was used to determine the kinetic parameters:

(14) $$\alpha \left(t\right)=A_{\alpha }\cdot e^{\frac{{-E}_{a}}{RT_{\alpha ,j}}}\cdot t$$

The degree of cure is expressed as a function of time α(t). Taking the natural logarithm of Equation (14) yields the following expression:

(15) $$\ln(t_{\alpha })=\ln\left[\frac{\alpha (t)}{A_{\alpha }}\right]+\frac{E_{a}}{RT_{\alpha ,j}}$$

The initial component of the right side of Equation (15) and the apparent activation energy can be derived from the linear correlation of $\ln(t_{\alpha })\;$and 1/*T*_j_ [36]. The apparent activation energies and the pre-exponential factors can be calculated using a linear regression according to Friedman by recording the temperatures and times for the corresponding degrees of cure. For each value of the degree of cure, the natural logarithm of the reaction time is plotted against the reciprocal of the temperature and a linear dependency is fitted. This is shown by the following relationship [36]:

(16) $$m=\frac{E_{a}}{R}$$

Also, the pre-exponential factor is calculated using the *y*-axis intercept corresponding to the following relationship [36]:

(17) $$y_{0}=\ln\left(\frac{A}{a}\right)$$

All DEA kinetic models depicted in this work were determined according to the above-discussed procedure.

### 2.4. Differential Scanning Calorimetry (DSC)

DSC measurements were performed with a DSC 204F1 Phoenix^®^ (NETZSCH-Gerätebau GmbH, Selb, Germany) with an integrated auto-sampler. All measurements were conducted under nitrogen atmosphere with a N_2_ flow rate of 40 mL/min. For each measurement, about 19.5 ± 1.0 mg of the unmolded material was weighted into aluminum crucibles (Concavus Pan And Lid From Al, NETZSCH-Gerätebau GmbH, Selb, Germany). The samples were cooled down to −60 °C, heated up via different heating rates of 2, 5, 10, 15, and 20 K/min to 260 °C. All DSC experiments were repeated three times. The changes in enthalpy were recorded and analyzed using Proteus Thermal Analysis software (NETZSCH-Gerätebau GmbH, Selb, Germany, Version 7.1.0).

The degree of cure (α) directly correlates with the measured heat flow (∆H) during the reaction of the raw material.

(18) $$\alpha_{t}=1-\frac{∆H_{t}}{{∆H}_{total}}$$

where *α*_t_ represents the degree of cure at a specific time, ∆*H*_t_ is the overall released heat at a specific time, and ∆*H*_total_ corresponds to the overall released heat during the complete reaction.

The collected data from the DSC measurements were then investigated using the program “Kinetic Neo” (NETZSCH-Gerätebau GmbH, Selb, Germany, Version 2.1.2.2). The isothermal models according to the Friedman method were then applied to interpret the data. The involved determining parameters such as activation energy and pre-exponential factor, offering a comprehensive understanding of the EMC curing. The Friedman approach provides essential insights into the intricate isothermal behavior of the epoxy matrix composites.

In addition to the kinetic analysis, time–temperature–transformation (TTT) diagrams were constructed. These diagrams were built upon previously determined glass transition temperature data obtained from reference sources [41]. This dual approach, combining kinetic modeling with TTT diagrams, contributes to a comprehensive characterization of the materials’ behavior under varying conditions.

## 3. Results and Discussion

As already mentioned, 5 min was the amount of time selected for the kinetic analysis of the materials. For this purpose, curing experiments at three different temperatures were carried out, each with three different injection speeds. Every experiment was repeated five times. A time period of 5 min was selected to reach the plateau of the ion viscosity curve and the associated end of the reaction. The selected temperatures are all within the possible process window. Below 160 °C, the parts cannot sufficiently be demolded, and above 190 °C, degradation by thermal decomposition occurs during the molding process in the additional presence of oxygen. For a brief overview, only one of the measured injection speeds (1.0, 2.5, or 4.0 mm/s) is shown, as well as the resulting modelling curves. For all injection speeds, a similar behavior is observed, with the same trends for higher temperatures, and the same results for the modelling are observed. Therefore, only the behavior for 2.5 mm/s injection speed is shown. The corresponding representative diagrams are listed in the appendix.

### 3.1. Kinetic Analysis via Dielectric Analysis (DEA)

**Figure 4:**
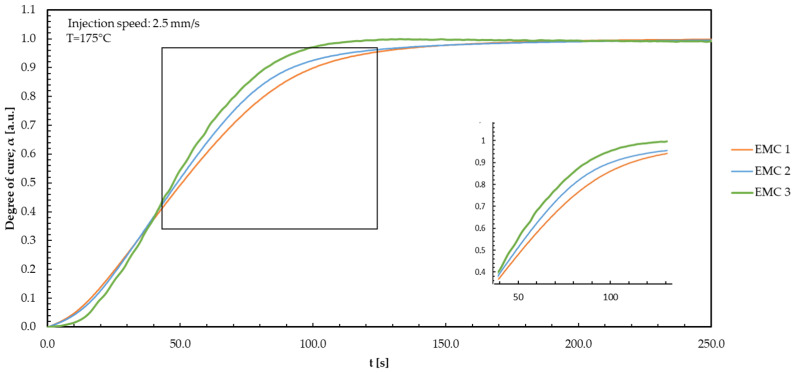
The degree of cure is plotted against the time during the TM process with 2.5 mm/s injection speed for the temperature 175 °C. The differences between EMC 1 (orange); EMC 2 (blue); and EMC 3 (green) are shown.

In the following, the results for the thermoset materials are shown. For a brief overview, only one of the measured injection speeds (1.0, 2.5, or 4.0 mm/s) is shown, as well as the resulting modelling curves. For all three injection speeds, similar behavior between the different temperatures and an adjusted *R*² of 0.988 for EMC 1; *R*² of 0.996 for EMC 2; and *R*² of 0.994 for EMC 3 are observed. The modelled curves, displayed in Figure 4, result from the model-free kinetic investigations. First, the collected temperature-compensated DEA signals are discussed, and afterwards, the results for the model-free kinetics are presented for each material.

The kinetic analysis of EMC 3 has already been conducted in preliminary studies; therefore, it is omitted in this paper [36,37].

As expected, the fast reaction reagent of EMC 3 has a significantly higher reaction rate and, like EMC 1, reaches the plateau of the DEA curve after 80 s and thus the end of the kinetically driven reaction. EMC 1 and EMC 2 show no differences in the first 18 s. In the further course, EMC 2 shows significantly faster conversion behavior and thus a higher reaction rate. The significantly higher reaction rate of EMC 2 leads to faster completion of the reaction and to the expected plateau of the DEA curve being reached earlier. In comparison, EMC 3 shows a significantly higher reaction rate between 0 and 45 s than EMC 2 and achieves higher degrees of cure up to this point. After 70 s, the reaction of EMC 2 starts to slow down and forms the expected reaction plateau. Therefore, EMC 2 reacts faster and reaches the maximum in ion viscosity earlier than EMC 1 and EMC 3. The heightened mobility of organic molecules in the compound promotes collisions and interactions among the reactants. The lower silica particle content also enhances collisions and interactions [12]. As a result, such factors significantly expedite the reaction progress.

**Figure 5:**
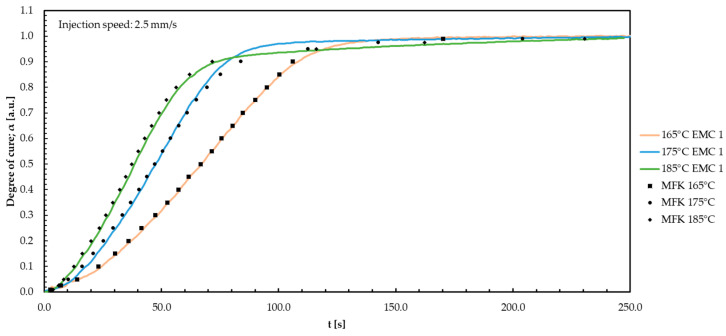
The degree of cure for EMC 1 is plotted against time during the TM process with an injection speed of 2.5 mm/s for the three temperatures. The model-free kinetic (MFK) modelling curves are shown in black.

Using Equation (10) from Section 2.3, model-free kinetic (MFK) curves can be generated for various temperatures. The MFK curves corresponding to temperatures of 165, 175, and 185 °C are discussed together with the measured DEA curves. As mentioned above, only the MFK for an injection speed of 2.5 mm/s is presented. The kinetic analysis of the materials was based on five repetitions for each temperature. The results for EMC 1 are discussed in Figure 5, followed by an analysis of EMC 2.

The modelling for EMC 1 agrees for the temperatures of 165, 175, and 185 °C. The deviation in the slope and inclination of the prediction curve to the measured data at 175 °C results from a higher degree of cure at 175 °C than at 165 °C and 185 °C. A linear calculation according to Friedman therefore deviates from the 0.85 conversion. This affects the pre-exponential factor and activation energy obtained by the kinetic equation. Examining Figure 5 illustrates that the modeling of MFK is based on a linearity between 165 °C and 185 °C. This assumption is generated using the model-free kinetic variant according to Friedman and leads to a divergent modeling approach for 175 °C. Similar behavior is observed for all injection speeds, representing the same trends for higher temperatures and the same results. The regression coefficient *R*^2^ of 0.988 for the MFK models indicates an accurate representation. The high correlation coefficient demonstrates a strong relationship between the actual measured data and the prediction models. The models are suitable for all measured injection speeds predicting the ionic viscosity and the corresponding degree of cure at 10 °C above and below the measured range, equivalent to a range of 155–195 °C.

**Figure 6:**
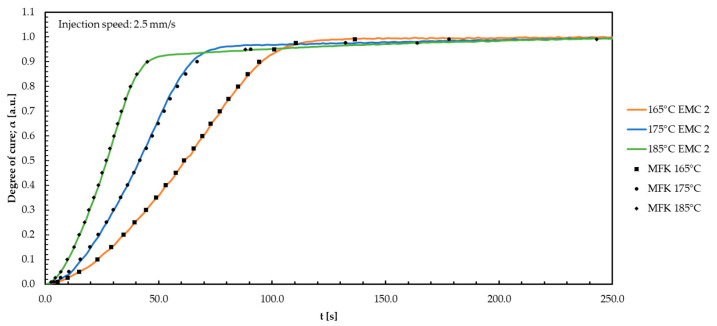
The degree of cure for EMC 2 is plotted against the time during the TM process for the three temperatures. The model-free kinetic (MFK) modelling curves are shown in black.

The same modelling for the three temperatures (165, 175, and 185 °C) matches with the results gained from EMC 2 as shown in Figure 6.

The same differences in the slope and the gradient of the curve for 175 °C EMC 1, caused by the linearity approach according to Friedman, as mentioned for EMC 1, is observed. All measured injection speeds show the same behavior in terms of temperature and injection speed changes.

For EMC 2, a higher regression *R*^2^ of 0.997 is noticed, indicating greater accuracy of the MFK models. Therefore, high-precision models for all measured injection velocities are applicable to predict the ion viscosity and the corresponding degree of cure in the range of 155–195 °C. The reduced silica particle content causes reduced electrical shielding and produces a significantly improved signal at reduced noise. The repeatability of the signals consequently increases, leading to a more accurate model.

### 3.2. Kinetic Analysis via Differential Scanning Calorimetry (DSC)

**Figure 7:**
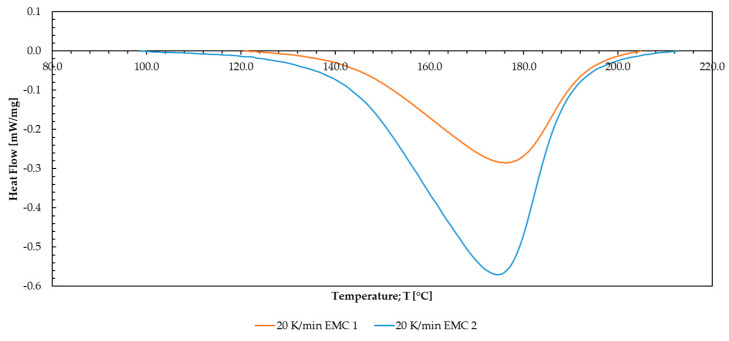
The results for the measured heating rate of 20 K/min of the investigated EMC 1 and 2 are plotted as heat flow against temperature.

For the investigated materials, large differences between the chosen heating rates (2, 5, 10, 15, and 20 K/min) are observed. In order to illustrate the differences, the results are displayed in Figure 7 for the heating rate of 20 K/min exclusively. Similar behavior is observed for the remaining heating rates (2, 5, 10, and 15 K/min) based on two repetitions of each measurement. Thermogravimetric analyses (TGAs) of the silica particle content were performed and calculated to normalize the differences to the raw mass for the establishment of kinetic models.

The reaction course depicted in Figure 7 shows that the curing reaction of EMC 1 takes place between 120 and 210 °C. For EMC 2, the reaction starts at around 100 °C and ends at around 230 °C. The exothermic reaction of EMC 2 is significantly higher, and the area below the peak is larger; 10 wt% less silica content similarly increases the organic content of the mass, leading to a larger exothermic value during reaction. Additionally, lower silica particle content causes decreased thermal isolation leading to an earlier reaction start. Furthermore, the higher organic content leads to a higher maximum value in heat flow and a bigger exothermic area.

**Figure 8:**
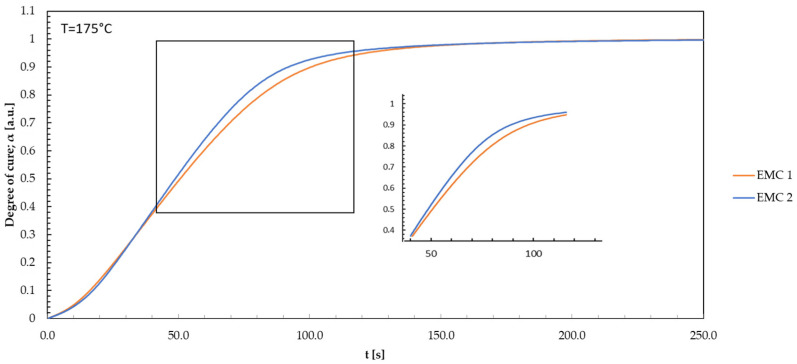
The degree of cure is plotted against time during the DSC process for EMC 1 and EMC 2 based on Friedman’s isothermal model-free kinetic model.

Equally, the DEA isothermal model-free kinetic models by Friedman were applied to evaluate the DSC-generated data. The conversion curves were calculated by linear integration of the exothermic curves, using a second heating as a baseline. This was used to perform Friedman MFK modeling. Using the isothermal prediction, the prediction curves for the temperatures used in the process can be plotted and compared to the DEA. Therefore, prediction models for the process temperatures of 165, 175, and 185 °C were created for EMC 1 and EMC 2. Since all temperatures selected exhibit comparable curve trends, only the curve for 175 °C is displayed in Figure 8.

Friedman’s isothermal model-free kinetic model exhibits a similar behavior to the models observed in the DEA modeling. EMC 2 demonstrates an enlarged slope in the first 30 s, surpassing the reaction rate of EMC 1 after 20 s in the area of 0 and 0.2 degree of cure. The effect becomes more pronounced in the range of 40 to 120 s and a degree of cure from 0.4 to over 0.9. Following the trend of EMC 2, a quicker response achieving the reaction endpoint is shown, corresponding to the conclusions of the DEA. The heightened mobility of organic molecules in the compound promotes collisions and interactions among the reactants. The lower silica particle content also enhances collisions and interactions [12,42]. As a result, such factors significantly expedite the reaction progress of EMC 2.

### 3.3. Comparison of DEA and DSC Kinetic Modelling

**Figure 9:**
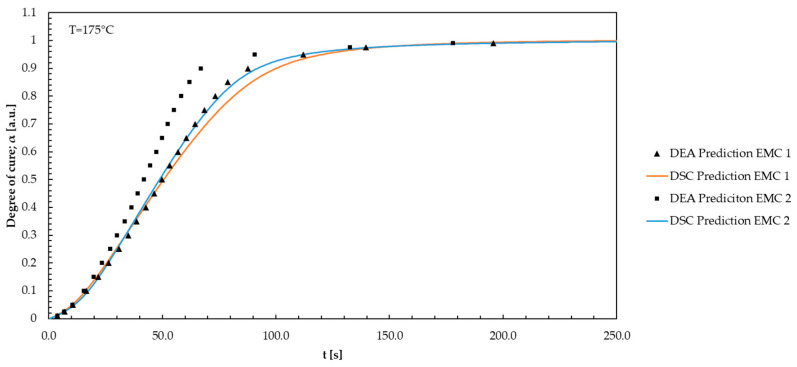
The degree of cure for EMC 1 and EMC 2 is plotted against time for Friedman’s model-free kinetic models evaluated with the DEA and DSC. Each model is represented for the temperature 175 °C.

To examine the analysis methods DEA and DSC, the degrees of cure for EMC 1 and EMC 2 are plotted against time for the Friedman’s model-free kinetic models obtained via DEA and DSC. Each model is displayed in Figure 9 for the temperature 175 °C.

The model curves exhibit no significant differences in the range between 0 and 10 s. Observation of the curves shows a significant deviation of EMC 1 after 18 s with a resulting faster rise of the curve standing for a faster reaction. After 40 s, differences in the DSC curves indicate the influence of the already discussed effects of the silica particle content. In the range from 130 s onwards, all prediction curves converge. The difference in kinetics between EMC 1 and 2 is significant. The DEA models represent major differences between the materials compared to the DSC models. All models show a high prediction accuracy of the kinetics with a regression coefficient of 0.988 for EMC 1 and 0.997 for EMC 2. It is important to note that the reaction parameters are not identical when comparing the two methods. The measurement methods differ in terms of the parameters and the system in which the measurements were carried out in order to create the respective model predictions. During DEA measurements, isothermal parameters were set in the cavity, where the mold pellet is rapidly brought to the desired isothermal process temperature directly with a high heating rate. In DSC measurements, samples are heated to the desired reaction temperature with comparatively low heating rates. The influence of the heating rate in the DSC can be roughly equated with the injection speed in the molding process. Despite the significant differences between these two measurement methods, high similarity in modeling for both used materials is still achieved.

Analyzing the DEA kinetics, the apparent activation energy was determined using the MFK iso-conversion methods as a function of the degree of cure curing. Equations (15) and (16) were used to perform the iso-conversion analysis.

**Figure 10:**
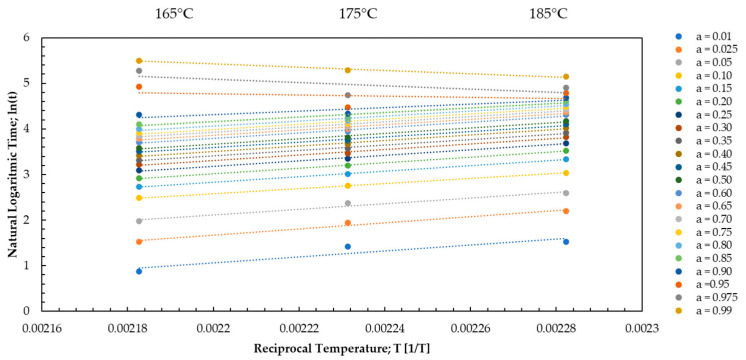
The natural logarithmic reaction time is plotted against the reciprocal temperature for an injection speed of 2.5 mm/s of each value for the degree of cure between 0.01–0.99 and the temperatures 165 °C, 175 °C, and 185 °C for EMC 1.

Temperature and time data were obtained during the measurement. The value for the activation energy and the pre-exponential factor was calculated from the data obtained during the measurement. For each value of the degree of cure, the natural logarithm of the reaction time is plotted against the reciprocal of the temperature as illustrated in Figure 10.

As for the DSC data, the model-free kinetic approach according to Friedman was applied similarly to the DEA kinetic evaluation. Here, conversion curves over time were determined by varying heating rates, and the apparent activation energy and pre-exponential factors for DSC were calculated using linear calculations according to Friedman, as described in Section 2.3. In the case of DEA, this was carried out within three temperature stages, resulting in three different apparent activation energies depending on the injection speed. All values for the DSC and DEA analyses for EMC 1 and EMC 2 are given in Table 1.

The values for the apparent activation energies *E*_a_ obtained via DEA for EMC 1 and EMC 2 given in Table 1 do not show any highly significant differences but are of comparable order of magnitude for all three injection speeds tested. However, they vary considerably between the two EMCs. While all values for EMC 1 are well below 60 kJ/mol, the values for EMC 2 are all in a range around 70 kJ/mol. Such differences in activation energies are not found on the basis of the DSC measurements. With DSC, the activation energy for both EMCs is practically identical at 65.5 kJ/mol for EMC 1 and 66.6 kJ/mol for EMC 2. DEA allows for detecting significant differences within all injection speeds with values of 54.9 to 72.2 kJ/mol (1 mm/s), 57.4 to 67.5 kJ/mol (2.5 mm/s), and 57.5 to 71.2 kJ/mol (4 mm/s). The absence of differences in the apparent activation energy determined via DSC is due to the normalization of the organic resin content as described in Section 3.2. In the determination of activation energy via DEA, no normalization is conducted for the organic fraction, which is why higher apparent activation energies are calculated with an increased organic content in EMC 2. The differences in the calculated *E*_a_ by DEA and DSC result from the difference in the basis of the calculation method, which was calculated linearly by the heating rate measurements in DSC and by the temperature measurements in the DEA. The range of measured values is in good agreement with values for *E*_a_ found in the literature data for the cross-linking of epoxies (50 to 90 kJ/mol) [43,44,45,46].

### 3.4. Time–Temperature–Transformation (TTT) Diagrams

**Figure 11:**
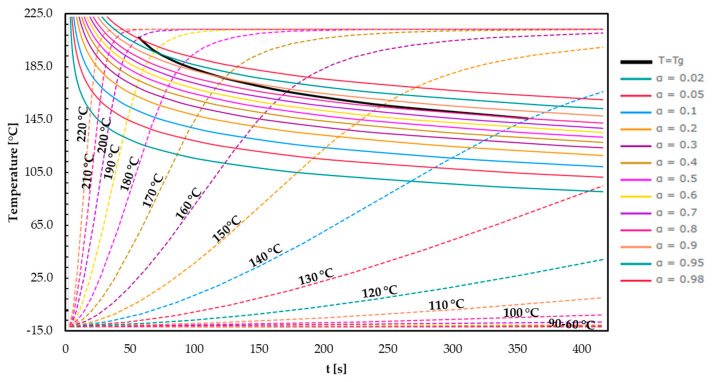
The time–temperature–transformation diagram plotted for EMC 1. The curves for the different degrees of cure (a = 0.02–0.98), the glass transition temperature for different isothermal temperatures (*T* = 100–220 °C), and the vitrification curve (*T* = *T*_g_) are showcased.

TTT diagrams were generated based on measured glass transition temperatures (*T*_g1_) of previous experiments and Friedman’s model-free kinetic models evaluated by DSC. Applying the well-established DiBenedetto equation, the construction of TTT diagrams makes it possible to gain deeper insight into the progress of the degree of cure and the glass transition temperature (*T*_g_) at different temperatures. Based on this analysis, TTT diagrams were designed for all three EMCs and consequently discussed. In Figure 11, the TTT diagram of EMC 1 is shown.

The *T*_g_ progression of EMC 1 is graphically represented in a TTT diagram as a function of temperature and time according to Section 1. The *T*_g_ progression curves for isothermal temperatures ranging from 100 to 220 °C and the degree of cure between 0.02 and 0.98 are depicted. The phase transition temperature for vitrification is indicated in black. The vitrification curve represents a boundary between the rubbery (under) and glassy (above) states. With increasing isothermal process temperature, a steeper slope in the *T*_g_ progression becomes apparent. Consequently, the corresponding degrees of cure are achieved at a later stage in the process. Comparing the time required to reach a degree of cure of 0.02 at process temperatures of 140 °C and 180 °C, it is noticeable that at 140 °C, the degree of cure is achieved after 260 s. In comparison, at 180 °C, the degree of 0.02 is reached after 50 s. Provided the curing temperature remains below *T*_g1_, an increase in the curing temperature results in a further enhancement in the *T*_g_ [47]. When examining the process parameter range (160–190 °C), the *T*_g_ closely matches the process temperature. At 170 °C, isothermally, the *T*_g_ reaches 175 °C in 120 s.

According to the literature, reactions continue to some extent beyond vitrification, leading to the glass transition temperature being higher than the cure temperature (*T*_g_ > *T*_g1_) [15,48,49]. Upon entering the vitrification phase, the molecular structure undergoes a shift from a gel to a glassy state, leading to a decrease in the movement of functional groups. Following this stage, the development of the reaction is mainly diffusion-controlled [50,51]. The TTT diagram demonstrates that the vitrification curve for EMC 1 at a process temperature of 170 °C is exceeded after 120 s, resulting in the system freezing with a degree of cure of 0.9.

At elevated temperatures approaching the final glass transition temperature (*T*_g1_), the reaction rate decreases further due to a low concentration of reactants at higher degrees of cure. Devitrification takes place as the cure temperature surpasses *T*_g_, resulting in the completion (*T*_g_ = *T*_g1_) of the reaction [15].

**Figure 12:**
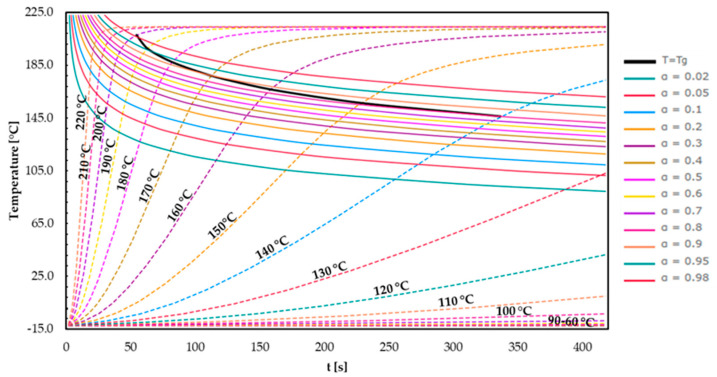
The time–temperature–transformation diagram plotted for EMC 2. The curves for the different degrees of cure (a = 0.02–0.98), the glass transition temperature for different isothermal temperatures (*T* = 100–220 °C), and the vitrification curve (*T* = *T*_g_) are showcased.

In Figure 12, the TTT diagram for EMC 2 is represented. 

The resulting main effects are similar to those in the evaluation of EMC 1. At a higher isothermal process temperature, the vitrification curve is reached more quickly. Using the same parameters, the vitrification curve at EMC 2 is reached earlier due to the accelerated kinetics identified in previous sections. At a processing temperature of 170 °C, a *T*_g_ of 175 °C is achieved within 107 s and a degree of cure of 0.9, primarily due to the higher organic content in EMC 2. EMC 2 is not able to reach the maximum T_g1_ even at a process temperature of 190 °C, whereby the material reaches a degree of cure of 0.95 and a *T*_g_ of 190 °C after approx. 60 s. The kinetic reaction is interrupted by reaching the glass transition point and only diffusion-driven reactions are possible, leading to a merely slower increase in *T*_g_.

**Figure 13:**
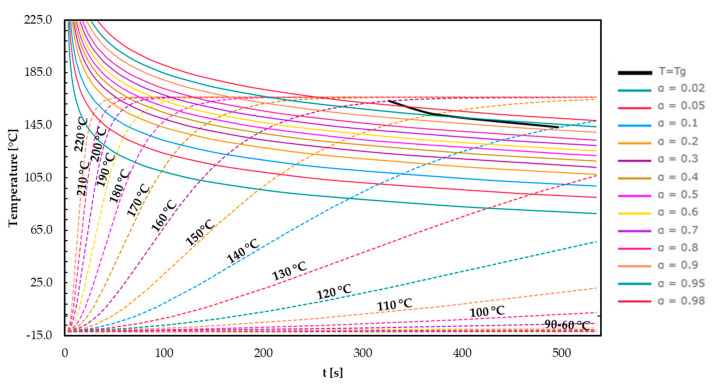
The time–temperature–transformation diagram plotted for EMC 3. The curves for the different degrees of cure (a = 0.02–0.98), the glass transition temperature for different isothermal temperatures (*T* = 100–220 °C), and the vitrification curve (*T* = *T*_g_) are showcased.

In Figure 13, the TTT diagram for EMC 3 is shown. 

EMC 3 represents a fast-curing material possessing a lower *T*_g1_ of about 168 °C, as illustrated in the obtained TTT diagram (see Figure 13). The time range on the time axis had to be extended to 550 s to illustrate the vitrification curve. Utilizing process temperatures above 170 °C, the maximum achievable *T*_g1_ = 168 °C is gained before reaching the vitrification curve, leading to a fully cured polymer network.

In summary, the TTT diagrams precisely illustrate the effect of a faster cross-linking of the mass with reduced silica particle content. In addition, the black *T* = *T*_g_ line indicates reaching the glassy state after a certain time and consequently achieving the end of kinetically dominated cross-linking. In order to verify the stop in reaction and possible re-crosslinking, post-mold cure (PMC) investigations are appropriate, which should prove a subsequent *T*_g_ increase.

## 4. Conclusions

In this study, the morphological properties in epoxy mold masses were monitored by assessing the degree of cure and the glass transition temperature (*T*_g_) by means of kinetic modelling based on DEA in-line process data. The in situ dielectric analysis (DEA) was compared to conventional offline differential scanning calorimetry (DSC). Materials with different reaction rates above and below *T*_g_ were used to generate time–temperature–transformation (TTT) diagrams, providing insights into the reaction’s behavior. Utilizing Friedman kinetic models proved to be a dependable method for predicting ion viscosity at specific stages during the curing process. The uniform success across all injection speeds enables the formulation of models that are independent of injection speed. Furthermore, confirming a correlation between glass transition temperature (*T_g_*) and ion viscosity (*IV*) creates the opportunity to extend the model for predicting the *T_g_* progression concerning process parameters.

The reproducibility results of the DEA signal quantified by the ion viscosity showed consistent results when the process parameters are kept constant, thereby demonstrating that DEA is a reliable in situ measurement technique for monitoring the cure progression of epoxy molding compounds in a real-world processing environment.

## Figures and Tables

**Table 1:** The apparent activation energy is shown for each injection speed for the DEA and DSC experiments in comparison of EMC 1 and EMC 2.

Material	DSC	DEA 1.0 mm/s	DEA 2.5 mm/s	DEA 4.0 mm/s
EMC 1	65.5 ± 1.7	54.9 ± 1.8	57.4 ± 1.6	57.5 ± 1.4
EMC 2	66.6 ± 2.3	72.2 ± 2.9	67.5 ± 1.5	71.2 ± 1.5

**Figure A1:**
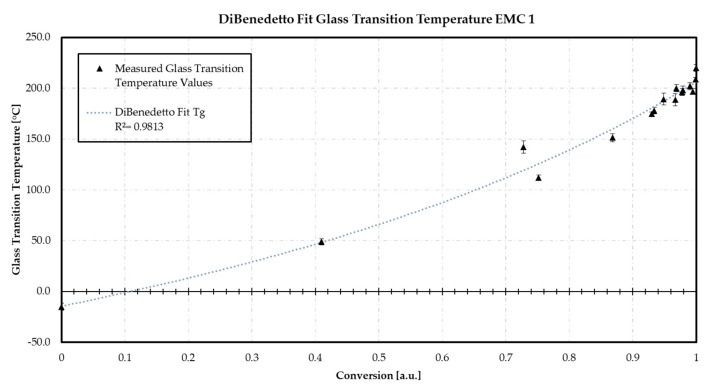
DiBenedetto fit utilized for the calculation of λ and the conversion of the already precured EMC 1.

**Figure A2:**
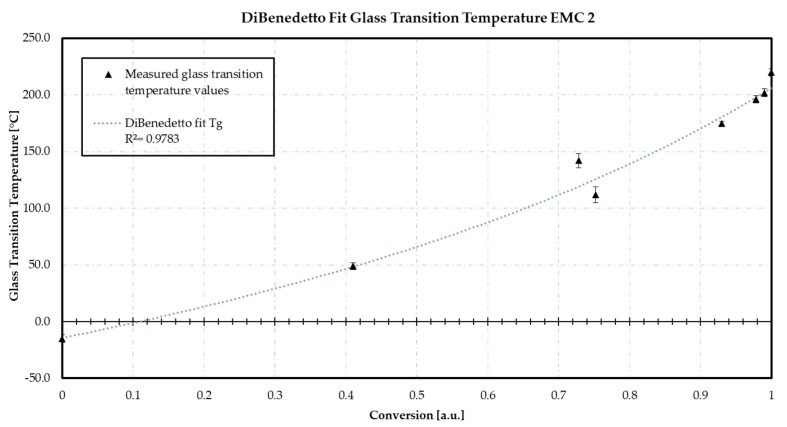
DiBenedetto fit utilized for the calculation of λ and the conversion of the already precured EMC 2.

**Figure A3:**
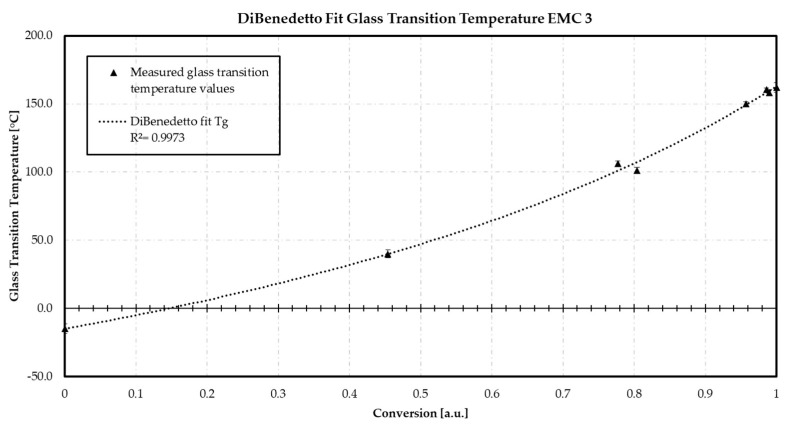
DiBenedetto fit utilized for the calculation of λ and the conversion of the already precured EMC 3.

**Figure A4:**
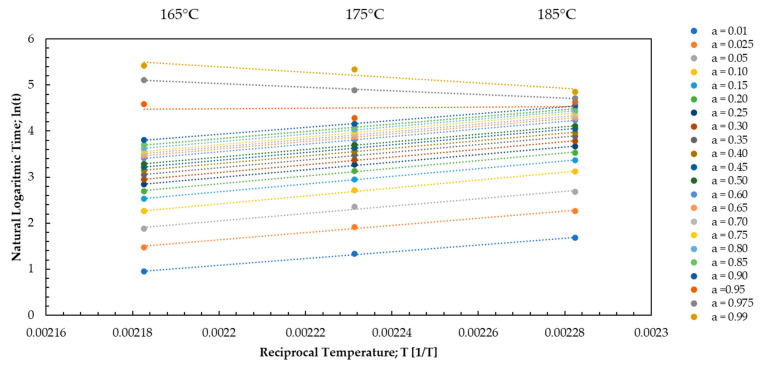
The natural logarithmic reaction time plotted against the reciprocal temperature for an injection speed of 2.5 mm/s of each value for the degrees of cure between 0.01 and 0.99 and the temperatures 165 °C, 175 °C, and 185 °C for EMC 2.

**Figure A5:**
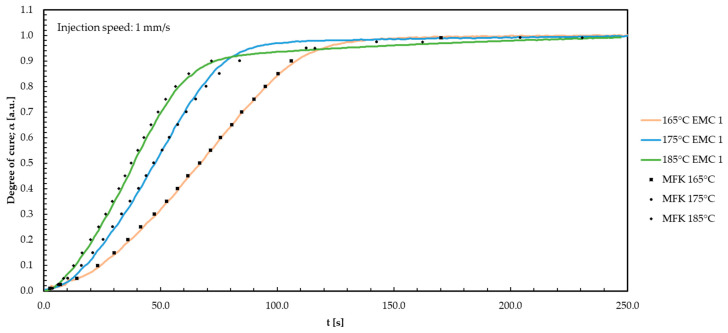
The degree of cure for EMC 1 plotted against time during the TM process with an injection speed of 1 mm/s for the three temperatures. In black, the model-free kinetic (MFK) modelling curves are shown.

**Figure A6:**
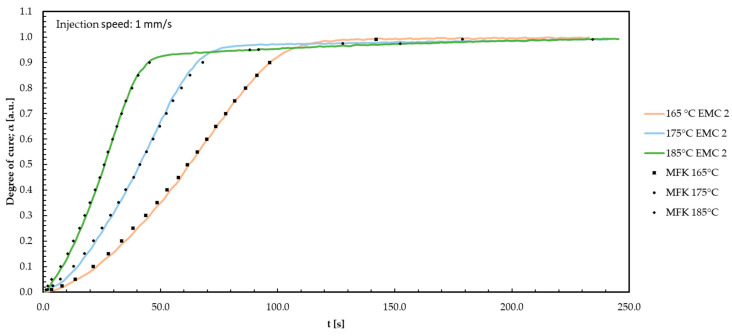
The degree of cure for EMC 2 plotted against time during the TM process with an injection speed of 1 mm/s for the three temperatures. In black, the model-free kinetic (MFK) modelling curves are shown.

**Figure A7:**
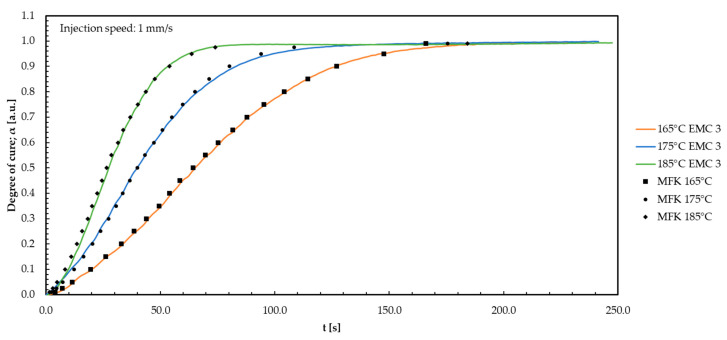
The degree of cure for EMC 3 plotted against time during the TM process with an injection speed of 1 mm/s for the three temperatures. In black, the model-free kinetic (MFK) modelling curves are shown.

**Figure A8:**
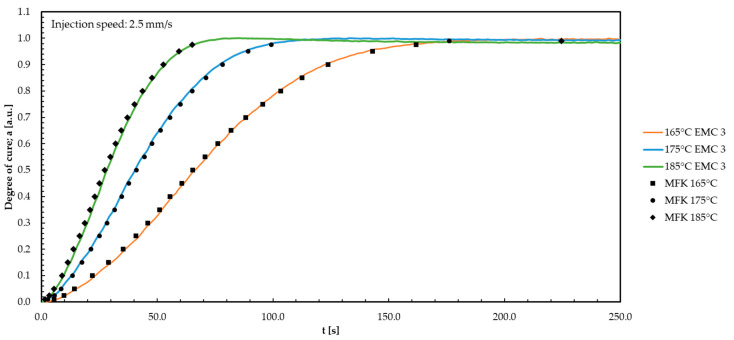
The degree of cure for EMC 3 plotted against time during the TM process with an injection speed of 2.5 mm/s for the three temperatures. In black, the model-free kinetic (MFK) modelling curves are shown.

**Figure A9:**
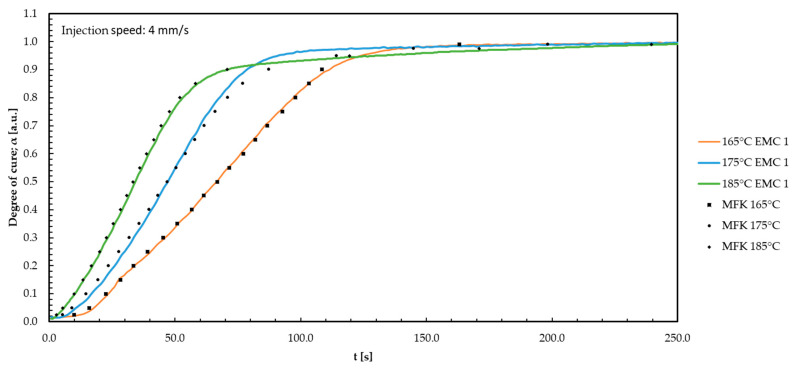
The degree of cure for EMC 1 plotted against time during the TM process with an injection speed of 4 mm/s for the three temperatures. In black, the model-free kinetic (MFK) modelling curves are shown.

**Figure A10:**
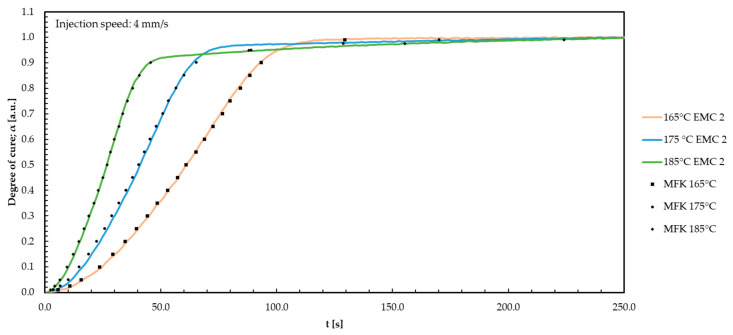
The degree of cure for EMC 2 plotted against time during the TM process with an injection speed of 4 mm/s for the three temperatures. In black, the model-free kinetic (MFK) modelling curves are shown.

**Figure A11:**
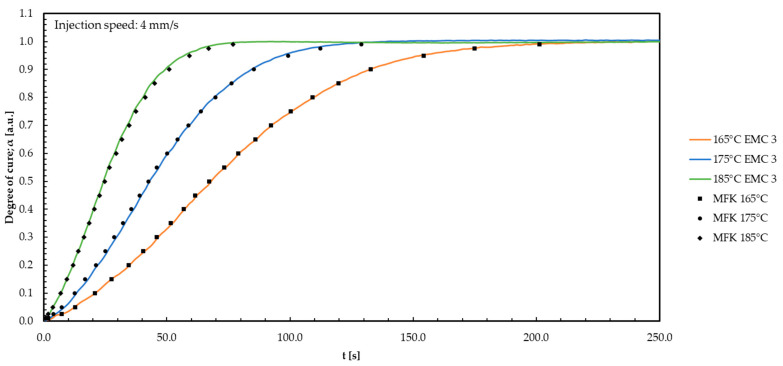
The degree of cure for EMC 3 plotted against time during the TM process with an injection speed of 4 mm/s for the three temperatures. In black, the model-free kinetic (MFK) modelling curves are shown.

**Figure A12:**
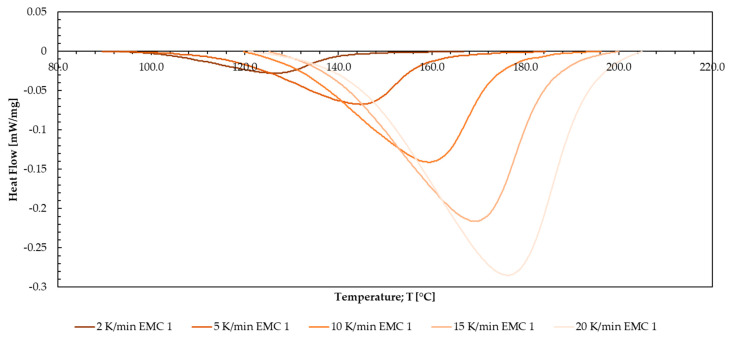
The results for the measured heating rates of 2, 5, 10, 15, and 20 K/min of the thermoset EMC 1 are plotted as heat flow against temperature.

**Figure A13:**
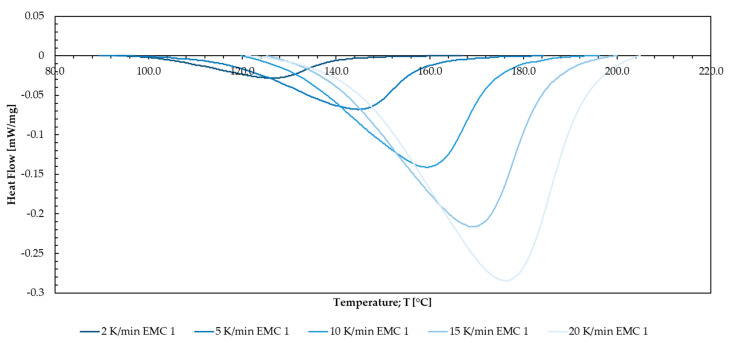
The results for the measured heating rates of 2, 5, 10, 15, and 20 K/min of the thermoset EMC 2 are plotted as heat flow against temperature.

**Figure A14:**
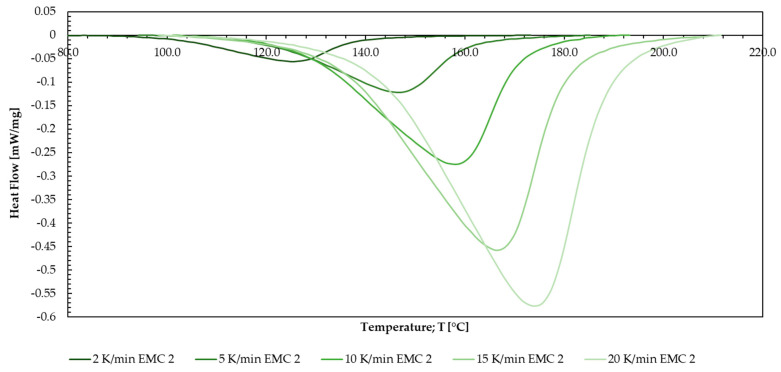
The results for the measured heating rates of 2, 5, 10, 15, and 20 K/min of the thermoset EMC 3 are plotted as heat flow against temperature.

**Figure A15:**
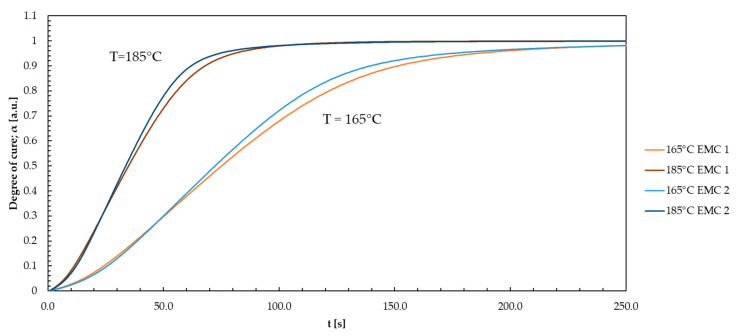
The degree of cure is plotted against time during the DSC process for EMC 1 and EMC 2 based on Friedman’s isothermal model-free kinetic model.

## Data Availability

Data are available upon request from the authors.
